# Who wants to be a chief veterinary officer (CVO)?–Thoughts on promoting leadership diversity in the public veterinary sector

**DOI:** 10.3389/fvets.2022.937718

**Published:** 2022-08-10

**Authors:** Katharina D. C. Stärk, Rosemary Sifford, Mary van Andel

**Affiliations:** ^1^Federal Food and Veterinary Office, Bern, Switzerland; ^2^United States Department of Agriculture, Washington, DC, United States; ^3^Ministry for Primary Industries, Wellington, New Zealand

**Keywords:** veterinary public health, veterinary officer, leadership, women in science, diversity

## Introduction

A Chief Veterinary Officer or CVO is a leadership role within a national Veterinary Service (VS). The title CVO in many countries implies legal responsibility for animal health, animal welfare and food safety for animal-derived food. The term CVO is also used for the delegates representing individual countries in the World Organization for Animal Health (OIE). In the context of this article, we use the term CVO as a placeholder for a leadership role within the veterinary public health community of a country. “Veterinary public health” is understood to cover all aspects of public health, i.e., the science of preventing disease, prolonging life and promoting health through the organized efforts of society (1), where veterinary knowledge is required. We apply a broad definition of public health that includes the more indirect impact of animal health (e.g., economic impact) and animal welfare on human well-being.

Based on correspondence with OIE staff, it becomes clear that only around a quarter of CVOs are women. This is consistent with low numbers of female leaders in other parts of the veterinary medicine sector such as professional organizations (2). This is in stark contrast to the composition of the student body enrolling for the veterinary degree. In most western countries, veterinary medicine has become a feminized profession, i.e., a profession where the gender balance is shifted from male to female (3). While in the 1970's, 16.8% of veterinary graduates in the US were female, this shifted to 44.3% in the 1980's and reaching 65.8% in 1996 (4). In the last survey of the veterinary profession in Europe, 58% of the respondents were female (5).

The question we want to discuss here is the following: Why is it that – out of all these female veterinary students – and at a time in history when women have more access to leadership roles than ever before, do so few pursue a career leading to a leadership role such as being a CVO?

## What does it take to become a veterinary public health leader?

In some countries, the only formal requirement to become a CVO (or comparable senior veterinary public health leader) is to have completed a veterinary degree plus a number of years of relevant experience, while in others, the requirements are specified in more detail [e.g., for the European Union, see (6)], listing the knowledge, skills and competencies required to be eligible to act as an Official Veterinarian and thus – ultimately – as a CVO.

Admission to veterinary degree courses tends to be highly competitive in many countries and access is based on merit-focused indicators which favors entry for women. There appears to be a difference in the motivation of female candidates compared to male (7). Over the past 20 years, the veterinary profession has become increasingly feminized (8). Once a profession becomes dominated by females, this is typically associated with the loss of status in society, e.g., in terms of remuneration (2). This then reduces its attractiveness to male candidates, thus resulting in a vicious cycle. In the case of veterinary medicine, increased numbers of female graduates have not translated into more women in academic positions, particularly above associate professor level (9, 10).

At postgraduate level, veterinary public health (VPH) is the specialty field with most relevance to the role of a CVO. VPH is a broad field including aspects of animal health, animal welfare, economics, ethics and legislation. VPH specialists are employed by government veterinary services, by industry, non-government organizations or as consultants. Specialization in VPH can thus be an attractive alternative to clinical specialization for women who plan for a family. Working hours can be more predictable compared to clinical duties including emergency rosters. More importantly, a role in VPH can be perceived as being valuable for society, it is associated with teamwork and with diverse and challenging problems, seen as distinctly different from routine clinical work (Gabrielle Bischopps, personal communication). There is evidence from other science-based disciplines that women tend to be more motivated by the opportunity to make a difference in an area they are passionate about than simply by financial gain or acclaim (11, 12). Based on the list of VPH specialists recognized by the European Board for Veterinary Specialization (EBVS) the female to male ratio among its members is almost equal (75:88; Andreas Wunsch, personal communication). The ratio amongst members in the Australia and New Zealand College of Veterinary Scientists VPH chapter is one woman to three men (17:51) and in the Epidemiology Chapter, one woman to almost two men (94:164) (Allen Petrey and Skye Freuen, personal communication). These numbers do not yet reflect the gender balance of the intake to veterinary schools which - in some cases - can be as high as 4 female students to 1 male or more.

However, becoming a CVO does not only require technical expertise, but it also implies leadership aspiration. While women earn an equal share of university degrees in general, women are under-represented in almost all sectors at senior management levels in most western countries (13). In the US, 40% of veterinary practices are under female management (14). On average, female veterinarians earn less than their male colleagues (5). A recent study by Smith (15) with earning data from veterinary practices in the US showed an unadjusted earning gap of 44%. About half of the earning differences cannot be explained by differences in working hours or other factors known to impact on practice productivity. There are more women in leadership positions in the public sector but even there, they remain underrepresented at senior level (see CVO numbers above).

Moving up the career pathway is linked to an organization's recruiting and progression policies. If you believe that gender discrimination is a thing of the past, you are probably suffering from the so-called “blind-spot bias” (16). This is the tendency of people to see themselves as less prone than others to unconscious influences, i.e., to cognitive bias. While most organizations nowadays acknowledge the value of diversity to foster innovation, creativity, adaptivity and performance, women remain under-represented in management positions. This finding remains even though key leadership traits such as extraversion, openness and conscientiousness are known to be common among women (17). Women are on the one hand, overrepresented in the traits that make up ‘transformational leadership' profiles and yet, paradoxically continue to be under-represented at senior leadership level (18). Many managers may reply that women in general lack the ambition to take on more responsibility and that their programmes for promoting women have failed to show impact. One reason for this is that ambitious women are a “norm violation.” Assertive women are perceived to be bossy, competent women as cold. No wonder it takes encouragement to aspire to roles associated with such characteristics. This is further aggravated by the fact that female vets are often suffering from the so-called impostor syndrome. This term refers to the tendency to doubt one's abilities despite contrary evidence. In a study conducted by Kogan et al. (19) among veterinarians, they found that feelings of self-doubt and fear of being “discovered as intellectual frauds” was particularly prevalent among female vets with only a few years of experience in practice and residing in New Zealand and the UK.

A lack of leadership ambition may also be due to practical reasons. As state childcare services vary between countries, female vets must often decide between career and family. This is despite the fact that veterinary training is among the most expensive degrees offered in western countries and often heavily subsidized by public funding. It should thus be of economic societal interest to retain as many women as possible in active veterinary employment.

In summary, while in principle, leadership career paths in veterinary public health should be attractive to female candidates given their over-representation at graduate level, the balance appears to shift at some point - resulting in an over-representation of male veterinarians in management roles. Similar observations are reported from the academic sector, a phenomenon referred to as the “leaky pipeline” (20).

## Discussion

The reasons for the lack of female leaders in a feminized sector such as veterinary medicine, and specifically in the public veterinary service, are diverse and not fully understood. Yet clearly, diverse teams have advantages, particularly in complex environments. It must therefore be desirable to foster diversity for all organizations and businesses that are serious about achieving their goals.

Based on our professional experience, there are no simple policies to “fix” the situation. From personal experience, we know that it is not enough to appoint one woman into a senior position to trigger a systemic change in work culture. To achieve progress, the first step is to confirm the fundamental value of diversity in leadership styles and career paths. There is not a single best profile for a senior veterinary manager; degrees are important but so is experience. Technical knowledge is essential, but so are skills in communication, negotiation, flexibility and improvisation. The commitment of a diverse leadership team is needed to bring about the change in culture that supports gender equality.

Governments and institutions that have acknowledged the value of gender balanced leadership have a variety of tools available to them to create the opportunities for attaining gender balance (21). These tools include policy development to both recruit and retain female leaders and communication of the success of these initiatives. These policies may include flexible working arrangements, active development of female talent, paid maternity leave, paid and unpaid childcare leave. It has been demonstrated that, over time, gender balanced leadership teams are able to undo previously biased and unequal access to organizational power (21).

Gender awareness training is an important first step which ideally should be started early as part of the veterinary curriculum, and then continued as part of continuing professional development (CPD) in universities and veterinary services. Students should be able to recognize and challenge discourse of limitation and discrimination before these become normalized, internalized and entrenched (22). Gender bias is a reality also in a profession where women are well-represented (23). Stereotypically expected behaviors do disadvantage both men and women. It is interesting to note that the stereotypical representations of leaders generally resemble stereotypes of men rather than stereotypes of women, though this is noted to be decreasing over time (18). It may not be surprising that CVOs are for the most part male given the breadth of experience required to attain the role, the perceptions of tradeoffs needed to be made with family time and the perception of how a CVO should conduct themselves.

Female role models are another important element to allow young women to imagine a future in which both professional aspirations and family planning can be realistically combined (24). Many veterinary schools are offering mentoring programmes where students can meet senior colleagues from different sectors to discuss career options. Even more powerful are female leaders that demonstrate not only the feasibility of career progression but also that this is a desirable path worth pursuing. Such inspiration and encouragement are essential not only for women, but also for men.

Many western countries are facing - or soon will be facing - a shortage of skilled workers. This offers opportunities for applicants; it strengthens their negotiation position. This offers a window of opportunity for women to specify the needs for change to make leadership attractive. The experience of the COVID-19 pandemic has opened new opportunities for flexible working that would not have been considered possible before. Dual income families will benefit from the technological solutions that increase independence of the physical location from which a job can be done.

We do not know enough about what stops women in the public veterinary service from seeking out leadership roles. Gender monitoring - a tool increasingly used by universities to document progress - would help strengthen the evidence base. Clearly, changing perceptions, values and practices is not an easy task; it takes energy, time and resources. Progress and output may not be tangible for some time. As with financial investment, believing in a future return is essential and to realize that others are pursuing the same goal may thus help strengthen our commitment. Here, we have made observations and suggestions based on our experience and on the findings of research in other disciplines. As we continue to mentor and guide a more diverse generation of future veterinary leaders, we look forward to more female veterinary professionals to pursue leadership roles. Key motivators for leadership roles are to gain more control over one's own time and life and to enhance the working lives of others (25). Mentoring can encourage individuals to explore alternative futures; mentoring is both effective and simple. It only takes a mentor that is willing to share advice and a mentee who is interested in learning from someone else's experience. For more systemic change, we need to intentionally examine and articulate the current state and identify and address those factors that limit diversity. External enablers such as leadership training and regular articulation and debate on diversity issues are important drivers of change (25). Thus, we can gradually realize the benefits of a diverse leadership culture, both for individuals and for the wider profession.

## Author contributions

The authors jointly developed the concept for this opinion. The first draft was prepared by KS, with RS and MA adding and revising the text. All authors contributed to the article and approved the submitted version.

## Conflict of interest

The authors declare that the research was conducted in the absence of any commercial or financial relationships that could be construed as a potential conflict of interest.

## Publisher's note

All claims expressed in this article are solely those of the authors and do not necessarily represent those of their affiliated organizations, or those of the publisher, the editors and the reviewers. Any product that may be evaluated in this article, or claim that may be made by its manufacturer, is not guaranteed or endorsed by the publisher.
